# Genomic insights into the population structure and genetic diversity of Ugandan indigenous cattle

**DOI:** 10.1111/age.70050

**Published:** 2025-10-27

**Authors:** Rodney Okwasiimire, Donald R. Kugonza, Daniil Ruvinskiy, Melak Weldenegodguad, Nasser Ghanem, Mahlako L. Makgahlela, Catarina Ginja, Richard P. M. A. Crooijmans, Juha Kantanen, Pekka Uimari, Kisun Pokharel

**Affiliations:** ^1^ Department of Agricultural Sciences University of Helsinki Helsinki Finland; ^2^ Natural Resources Institute Finland Jokioinen Finland; ^3^ Department of Animal and Range Sciences, College of Agricultural and Environmental Sciences Makerere University Kampala Uganda; ^4^ Department of Animal Production Cairo University Cairo Egypt; ^5^ Animal Breeding and Genetics Agricultural Research Council Pretoria South Africa; ^6^ Department of Animal, Wildlife and Grassland Sciences University of the Free State Bloemfontein South Africa; ^7^ Program in Genomics, Biodiversity and Land Planning, CIBIO, CIISA, Faculty of Veterinary Medicine University of Lisbon and BIOPOLIS Vairão Portugal; ^8^ Animal Breeding and Genomics Wageningen University and Research Wageningen The Netherlands

**Keywords:** African native cattle, hybridization, livestock genetics, phylogenetics, population characterization, whole‐genome sequencing

## Abstract

Domestic cattle in Africa can be categorized as either taurine (*Bos taurus*) or indicine (*Bos indicus*) based on their domestication histories from the extinct aurochs (*Bos primigenius*). Close to 150 breeds of indigenous cattle are estimated to exist in Africa and have a complex mixture of *B. taurus* and *B. indicus* ancestries. Native cattle in Uganda fall into three broad categories: the Sanga, East African Shorthorn Zebu, and Zenga. There is limited information about the genetics of Ugandan indigenous cattle, despite their representation of nearly 80% of the national herd. In the present study, we describe the genetic diversity and population structure of five native breeds using whole genome sequences of 95 animals. For a comparative context, we included published whole genome sequences of 97 animals in the analysis. Our findings indicate a clear distinction between Zebu, Sanga, and Zenga breeds, with elevated inbreeding and lower genetic diversity levels among the Ugandan breeds. We also observed an introgression of European genetic resources into Ugandan native cattle breeds. Notably, our results suggest existence of two subpopulations within the Nganda breed, a finding that has implications on the conservation efforts of native animal genetic resources. The findings of this study show that indigenous cattle genetic resources in the country are threatened by admixture with imported genetic material and underscore the need for targeted efforts to characterize and conserve them before they are lost to crossbreeding and breed substitution.

## INTRODUCTION

Indigenous cattle in Africa have a complex history and are believed to have been introduced to the continent in three waves, via present‐day Egypt (the Nile Valley) or Somalia (the Horn of Africa) (Epstein, 1957; Hanotte et al., 2002). These introductions followed domestication events of taurine cattle (*Bos taurus*) in the Fertile Crescent (around present‐day Iran, Iraq, and Turkey) and the indicine (*Bos indicus*) in the Indus Valley (present‐day Pakistan) (Ajmone‐Marsan et al., 2010). Additional domestication events for cattle in Africa, Europe, and Asia have also been put forward (Bradley et al., 1996; Lei et al., 2006; Marshall & Hildebrand, 2002). Native cattle in Africa fall into three broad categories: (i) the humpless taurine (*B. taurus*); (ii) the humped Zebu (*B. indicus*); and (iii) the Sanga (*Bos africanus*). Sanga cattle have characteristic long and slender horns and other intermediate features of both humpless taurine and humped Zebu (Grigson, 1991). A different classification system categorizes native African cattle into four general categories. These include the humpless cattle of West and Central Africa (*B. taurus*); the humped cattle (Zebu) of East and parts of West Africa (*B. indicus*); the Sanga cattle of East and South Africa; and the Zenga (Sanga × Zebu) cattle of East Africa (Rege, 1999). The Sanga are believed to have arisen following crossbreeding between the first Zebu cattle on the African continent which were introduced around 1500 BC, and humpless longhorn cattle introduced around 5000 BC. The Zenga cattle emerged through crossbreeding between the Sanga and Zebu introduced during the second wave of cattle migration around 670 AD (Epstein, 1957).

Native cattle represent about 77% of Uganda's total herd, which has approximately 14.5 million animals (UBOS, 2024). Cattle are kept as sources of income (social capital, insurance, and savings), food, draft power, and other goods and services. These contribute over 40% to the value of livestock production and 4.3% to the country's GDP (FAO, 2019). The majority of cattle in Uganda are reared in the cattle corridor; a stretch of over 40 districts that covers close to 35% of the country's land mass from the southwestern to northeastern parts of the country (Kiggundu et al., 2019; Tayebwa et al., 2018). The animal husbandry practices in this stretch are mainly pastoral and agro‐pastoral, with conditions characterized by episodes of seasonal pastures, water scarcity, and heavy parasite infestation including ticks and tsetse flies. Despite these challenges, the cattle corridor is preferred by native cattle keepers. This predilection is due to the corridor's vast rangelands and savannah grasslands that support the pastoral system of grazing, where animals search for both pasture and drinking water (Egeru et al., 2015). As a result, indigenous cattle breeds such as the Ankole, East African Shorthorn Zebu (EASZ), and Nganda, that are known for their resilience are preferred (Kasaija et al., 2021) and are kept by nearly 81% of cattle‐keeping households in the country (UBOS, 2024).

In most African countries, breeding approaches and institutional policies are often unstructured and in favor of highly productive exotic and crossbred animals (Nyamushamba et al., 2016). In Uganda, for example, the imported cattle population increased by 78.8% between 2008 and 2021 compared to an increase of 5.4% in the same period for native cattle (UBOS, 2024). About 32% of indigenous cattle breeds in Africa have been reported to be at risk of extinction. In Uganda, the Kyoga, Serere, Usuk, and Bahima strains of the Ankole breed have previously been classified as “threatened” due to conflicts, crossbreeding and interbreeding. A further 22 African breeds including the Kigezi shorthorn and Karagwe shorthorn breeds of Uganda were reported extinct (Rege, 1999). Consequently, the unique animal genetic resources possessed by native breeds could be lost even before they are well characterized. Previous research on characterization of Uganda's native cattle includes phenotypic studies (Kabi et al., 2015; Masaba et al., 2024; Ndumu et al., 2008) and use of microsatellite markers for the Ankole breed (Kugonza et al., 2011). In addition several researchers have co‐analyzed Ugandan breeds alongside other African cattle (Gebrehiwot et al., 2020; Hanotte et al., 2000; Kim et al., 2017; Nanaei et al., 2020; Taye et al., 2017), and featured Ugandan cattle in a resource of genome sequences of African breeds (Tijjani et al., 2024).

The objective of this study was to provide an in‐depth understanding of the population structure and genetic diversity of indigenous cattle breeds from Uganda using whole genome sequence data. According to the Domestic Animal Diversity Information System (DAD‐IS) of the Food and Agriculture Organization (FAO), nine breeds are native to Uganda (FAO_DAD‐IS, 2025). This study focused on five of them: the Ankole, Karamojong, Nganda, Nkedi, and Ntuku.

## MATERIALS AND METHODS

### Animals

**FIGURE 1:**
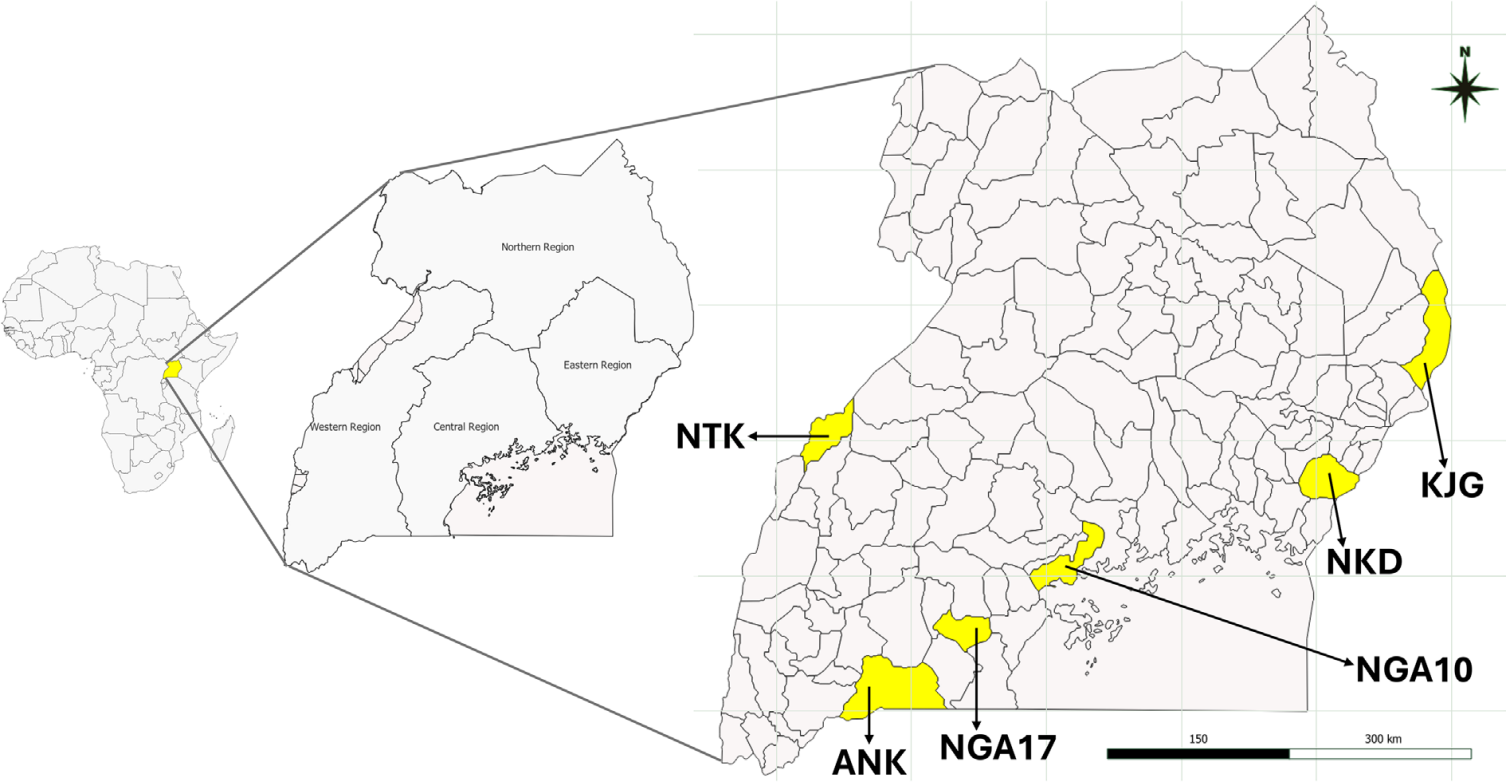
Geographical location of the districts from which samples of the native Ugandan cattle breeds analyzed in this study were obtained. NTK: Ntoroko (Ntuku breed), ANK: Isingiro (Ankole breed), NGA17: Lwengo (Nganda breed), NGA10: Mpigi (Nganda breed), NKD: Tororo (Nkedi breed), and KJG: Amudat (Karamojong breed). Figure S1 shows images of the different breeds.

Blood samples were collected from a total of 95 animals from four regions (and six districts) of Uganda during implementation of the LEAP‐Agri OPTIBOV project (grant number: 727715). The project aimed to characterize traditional (locally adapted) cattle breeds and use genetic variation linked with their adaptation to local ecosystems for improvement of breeding schemes for the future (production, longevity, disease resistance). These included the Central (*n* = 27, from Lwengo and Mpigi districts), Eastern (*n* = 19, from Tororo district), Northern (*n* = 11, from Amudat district), and Western (*n* = 38, from Isingiro and Ntoroko districts) regions (Figure 1). The sampled animals were from five breeds: Ankole (*n* = 19), Ntuku (*n* = 19), Nkedi (*n* = 19), Karamojong (*n* = 11), and Nganda (*n* = 27; Table 1, Tables S1, and S2, Figure S1). At each sampling site, at least one male animal was sampled, and only animals identified by the owner as purebred and unrelated were included. For comparative analysis of the population structure and genetic diversity, we downloaded publicly available sequences of 54 African indigenous cattle (nine each from six breeds) including Boran, Fulani, Kenana, N'dama, Muturu, and Ogaden. We also downloaded sequences of 21 European cattle (three breeds), including five Dutch Holstein Friesian, eight Dutch Deep Red, and eight Groningen White Headed. Moreover, we downloaded sequences of 22 Asian cattle (four breeds) including eight Dhanni, five Gir, four Nelore, and five Yakutian cattle (Table 1, Tables S1, and S3), all from the European Nucleotide Archive.

**TABLE 1:** Summary of the sequences analyzed in this study, including those downloaded from the European Nucleotide Archive.

Breed	Breed category	Breed code	Origin	Origin code	Bio‐project	Animals (*n*)
Boran^b^	*Bos indicus*	BRN	Africa	AFR	PRJNA312138	9
Fulani^b^	*B. indicus*	FLN	Africa	AFR	PRJNA858239	9
Kenana^b^	*B. indicus*	KEN	Africa	AFR	PRJNA312138	9
Muturu^b^	*Bos taurus*	MUT	Africa	AFR	PRJNA386202	9
N'dama^b^	*B. taurus*	NDM	Africa	AFR	PRJNA312138	9
Ogaden^b^	*B. indicus*	OGN	Africa	AFR	PRJNA312138	9
Dhanni^b^	*B. indicus*	DHN	Asia	ASN	PRJNA658727	8
Gir^b^	*B. indicus*	GIR	Asia	ASN	PRJNA277147	5
Nelore^b^	*B. indicus*	NEL	Asia	ASN	PRJNA277147	4
Yakutian cattle^b^	*B. taurus*	YAK	Asia	ASN	PRJEB28185	5
Deep Red^b^	*B. taurus*	DRD	Europe	EUR	PRJEB56301	8
Groningen White Headed^b^	*B. taurus*	GWH	Europe	EUR	PRJEB56301	8
Holstein^b^	*B. taurus*	HOL	Europe	EUR	PRJEB56301	5
Nganda^a^	*B. indicus* (Zenga)	NGA	Uganda	UGA	PRJEB90914	27
Nkedi^a^	*B. indicus* (Small East African Shorthorn Zebu)	NKD	Uganda	UGA	PRJEB90914	19
Karamojong^a^	*B. indicus* (Large East African Shorthorn Zebu)	KJG	Uganda	UGA	PRJEB90914	11
Ankole^a^	*B. indicus* (Sanga)	ANK	Uganda	UGA	PRJEB90914	19
Ntuku^a^	*Bos indicus* (Sanga)	NTK	Uganda	UGA	PRJEB90914	19

^a^ Novel sequence data from this study.

^b^ Public sequence data downloaded from the European Nucleotide Archive.

### DNA extraction and sequencing

Total DNA was extracted from blood samples using the salting out method (Miller et al., 1988). The presence and integrity of the extracted DNA was assessed by agarose gel electrophoresis while quantification and quality of the DNA was examined using a UV/Vis Spectrophotometer (Biochrom Ltd., Cambridge, England). Sequencing was done following the paired‐end (reads of 150 bases) strategy with single‐indexed genomic libraries on an Illumina NovaSeq 6000 Sequencing System (Illumina Inc., San Diego, CA, USA).

### Sequence data quality control and preprocessing

Quality analysis of the raw fastq files was done with fastqc v0.11.9 (Andrews, 2010) and reports were summarized and aggregated with multiqc v1.19 (Ewels et al., 2016). Sequencing adapters were removed from the raw reads with trimmomatic (v0.39) (Bolger et al., 2014) with default settings to generate clean reads prior to mapping to the reference genome sequence.

### Read mapping, variant calling, filtering, and annotation

The clean reads were processed following the Genome Analysis Tool Kit (gatk v4.6.0.0) best practices workflows for data pre‐processing for variant discovery (Van Der Auwera et al., 2013). Using bwa‐mem v0.7.17 (Li & Durbin, 2010) with default parameters (save for the ‐M option), reads were mapped to the *B. taurus* genome ARS‐UCD1.3 downloaded from Ensembl release 112 (Dyer et al., 2025). samtools v1.18 (Li et al., 2009) was used to convert the resulting SAM files to BAM format and for their subsequent sorting. Duplicates were marked and removed with MarkDuplicates tool in picard tools v3.1.1. The AddOrReplaceReadGroups tool of the same software was used to add read group information followed by the SortSam tool to sort the resulting file. Base quality score recalibration was performed with the BaseRecalibrator tool from GATK, incorporating a *B. taurus* known variants file downloaded from Ensembl release 112.

Variant calling and filtration were performed following GATK's best practice workflows for germline short variant discovery (SNPs + Indels) (Poplin et al., 2017). Briefly, we called variants from each sample with the HaplotypeCaller tool (in GVCF mode), and the resulting GVCF files for each breed were consolidated with the CombineGVCFs tool. This was followed by joint genotyping with the GenotypeGVCFs tool to yield jointly called variants ready for filtration. Variant filtration was then done to retain variants that passed parameters QD <2.0, QUAL <30.0, FS >60.0, MQ <40.0, SOR >3.0, MQRankSum <−12.5, ReadPosRankSum <−8.0 for SNPs and QD <2.0, QUAL <30.0, FS >200.0, and ReadPosRankSum <−20.0 for indels. Missing genotypes were recoded using bcftools (Danecek et al., 2021) and biallelic autosomal variants that passed the mentioned filters were selected with the GATK SelectVariants tool for downstream analysis.

In addition, missing genotypes in each sample and the extent of relatedness with other samples were respectively obtained using the ‐‐missing‐indv and ‐‐relatedness, functions in vcftools v0.1.17 (Danecek et al., 2011). The relatedness values for all samples were below 0.8, and no samples were eliminated on this basis. Similarly, no samples were eliminated due to missing genotype data (threshold set at 0.2). The thresholds were applied to exclude related individuals and samples with excessive missing genotypes, as population structure analyses (e.g., principal components analysis [PCA] and admixture) can misinterpret family relationships as true structure. Additionally, high rates of missing genotypes indicate poor‐quality sample DNA, which can produce unreliable genotyping results (Liu et al., 2020). Polymorphism statistics including the number of SNPs and indels, the transition to transversion ratio (Ts/Tv), and the heterozygous to homozygous (Het/Hom) SNP ratio were obtained using the stats function in bcftools v1.18 (Danecek et al., 2021).

The Ensembl Variant Effect Predictor (McLaren et al., 2016) was used to determine the effects and location of the detected variants for each breed following the *B. taurus* ARS‐UCD1.3 assembly and dbsnp v150 resources. Variants that were not present in dbsnp v150 were denoted as “novel” whereas those present in the database were denoted as “existing” variants.

### Population structure

To examine the population structure, linkage disequilibrium (LD) pruning (‐‐indep‐pairwise 50 10 0.2) was done in plink v1.90 (Chang et al., 2015). The parameters used considered a chromosome window of 50 SNPs, removing an SNP from a pair with an LD value higher than 0.2, and then moving the SNP window by 10 SNPs to repeat the process. This was done to reduce the number of variants with redundant data that could have otherwise caused misrepresentation of the genetic structure (Liu et al., 2020).

For PCA, eigenval and eigenvec for the 18 breeds were obtained with the ‐‐pca prompt in plink v1.90 and visualized with the ggplot2 v3.5.1 (Wickham et al., 2007) package in r v4.4.0. To ascertain the phylogenetic clustering of the entire dataset, a distance matrix was calculated from the VCF file using vcf2dis v1.53 (https://github.com/BGI‐shenzhen/VCF2Dis) with default parameters. A neighbor‐joining (NJ) phylogenetic tree was then constructed from the calculated distance matrix with the fastme v2.0 web server (Lefort et al., 2015), and visualized in iTOL (Letunic & Bork, 2024). In addition, a NeighborNet network was constructed from the *F*_ST_ matrix of the cattle populations using r package phangorn v2.12.1 (Schliep et al., 2008).

The ancestral populations among all the 18 breeds studied were inferred with admixture v 1.3.0 (Alexander et al., 2009). The software estimates ancestry among individuals using multilocus SNP data by maximum likelihood (ML) algorithms implemented through block relaxation approaches (Alexander et al., 2009). With each subcategory of cattle in the data set assumed as an ancestry (Asian, European, African taurine, Sanga, Zenga, East African Small, and Large Zebu ancestries), the software was set to estimate ancestral values (*K*) ranging from 2 to 7. Cross‐validation values (generated with the cv flag) for each assumed ancestral population were plotted against their corresponding *K* values with the ggplot2 v3.5.1 (Wickham et al., 2007) package in r v4.4.0. The lowest point on the plot was selected as having the ideal predictive accuracy. The resultant matrix of estimated fractions (*Q*) files from *K* values up to the ideal were plotted and visualized in r v4.4.0 with the pophelper v2.3.1 package (Francis, 2017).

Using the same pruned dataset, we investigated phylogenetic relationships between the cattle breeds following the ML algorithm implemented in treemix v1.13 (Pickrell & Pritchard, 2012) with blocks of 1000 SNPs (‐k 1000). The software conducts population‐level admixture using residual matrices to identify pairs of population groupings that are poorly fitting the predicted ML tree and adds migration edges to rearrange the tree and account for potential migration events. From zero (no migration), potential migration events are gradually added to the modeled tree until a 99.8% explanation of the variance observed in the data is achieved. We visualized the modeled ML trees and residuals in r v4.4.0 using the complementary plotting script provided in treemix.

### Genetic diversity

The variant density across the autosomal genome was evaluated in sliding window sizes of 10k, 25k, 50k, and 100k with the –SNPdensity function of vcftools v0.1.17 (Danecek et al., 2011). We considered a window size of 50k for subsequent analysis since all the windows had identical average variants per kb across all autosomes. The minor allele frequency and SNP missingness was evaluated with the ‐‐freq and ‐‐missing options, respectively, in plink v1.90 (Chang et al., 2015).

The SNPs were then filtered with parameters ‐‐maf 0.05 ‐‐geno 0.05 in plink v1.90. The nucleotide diversity (*π*) within each breed was estimated following the parameters ‐‐window‐pi 50 000 ‐‐window‐pi‐step 25 000 in vcftools v0.1.17 (Danecek et al., 2011). Nucleotide diversity estimation was carried out to identify the average number of polymorphisms between a given pair of DNA sequences in each breed (Kanaka et al., 2023).

In plink v1.90, the ‐‐hardy prompt was used for estimation of observed (*H*_o_) and expected (*H*_e_) heterozygosity. The ‐‐het and ‐‐freq prompts were used to calculate the excess of homozygosity based inbreeding coefficient (*F*_HOM_) and minor allele frequency, respectively. Runs of homozygosity (ROH) were estimated using plink v1.90 with the ‐‐homozyg command, applying the following parameters: ‐‐homozyg‐kb 500, ‐‐homozyg‐snp 50, ‐‐homozyg‐window‐het 2, ‐‐homozyg‐window‐threshold 0.04. The number and length of the ROH were then classified by size into small (0.5–1 Mb), medium (1–5 Mb) and large (>5 Mb) (Liu et al., 2022; Xu et al., 2019). The autozygosity inbreeding coefficient (*F*_ROH_) for each breed as derived from ROH was then calculated by dividing the total length of ROH detected in each animal by the length of the cattle autosomal genome (McQuillan et al., 2008). A value of 2 489 385.779 kb was the calculated length of the autosomal genome from this study's data. The extent of decay of LD as measured by *r* ^2^ between pairwise SNPs for each breed was assessed with PopLDdecay (Zhang et al., 2019) with default settings.

Weir and Cockerham's pairwise *F*_ST_ statistic (Weir & Cockerham, 1984) was calculated with options ‐‐*F*_ST_‐window‐size 50 000 and ‐‐*F*_ST_‐window‐step 25 000 in vcftools v0.1.17 (Danecek et al., 2011). For pairwise calculations, random subsampling was done for breeds with larger sample sizes to allow comparison of matching sample numbers. Similarly, Tajima's *D* statistic which is a combined evaluation of the nucleotide diversity, and the number of segregating sites (Tajima, 1989) was estimated with the ‐‐TajimaD 50 000 option in vcftools v0.1.17 (Danecek et al., 2011). The average values of pairwise *F*_ST_, nucleotide diversity and Tajima's *D* statistic weighted by the number of variants in each window for each breed were reported.

## RESULTS

### Sequencing, mapping, filtration, and annotation results

The 95 Ugandan cattle (5 breeds) had an average of 223 120 881 (± 17 815 919) reads mapped to the bovine genome assembly ARS‐UCD1.3. We obtained mapping statistics of depth 12.53× (± 1.02), mapping rate of 98.91 (± 3.42), and coverage rate of 96.52 (± 0.78; Tables S1 and S2). The downloaded public sequences from other breeds (13 breeds, 97 cattle) had an average of 156 190 041 (± 48 749 709) reads with a depth of 10.56× (± 1.89), mapping rate of 99.11 (± 0.52), and coverage rate of 98.29 (± 0.88; Tables S1 and S3).

Following GATK's best practices workflows for Germline short variant discovery (SNPs + Indels), an average of 10 289 820 (± 676 750) SNPs and 1 367 315 (± 81 449) indels were obtained per animal. The highest mean number of SNPs (11 255 505, ± 341 563) was recorded in the Karamojong breed while Ankole had the lowest (9 601 274 ± 209 592). The Nkedi breed had the highest mean number of indels (1 413 983 ± 63 235) while Ankole had the lowest (1 296 995 ± 33 038). The observed average transition/transversion (Ts/Tv) ratio was 2.26 ± 0.05 and the Het/Hom ratio was 0.18 ± 0.03 (Table S1). A total of 28 117 471 (17.09%) variants were novel among the Ugandan cattle breeds, with the Nganda breed having the highest (6 582 520) and the Karamojong (4 268 143) the lowest (Table S1). The observed difference in the number of variants per breed could be attributed to the varying numbers of genotyped animals for the two breeds (Nganda, *n* = 27 and Karamojong, *n* = 11; Tables S1 and S2). Variant Effect Predictor results showed that most of the annotated variants were either intergenic (46.02%) or intronic (44.38%; Table S4).

### Population structure

Overall, the 18 breeds collectively had 52 187 497 biallelic autosomal SNPs. After filtering according to missing data and minor allele frequency, 22 455 225 variants were extracted, and 1 428 484 variants were retained after subsequent LD pruning.

### Principal component analysis

**FIGURE 2:**
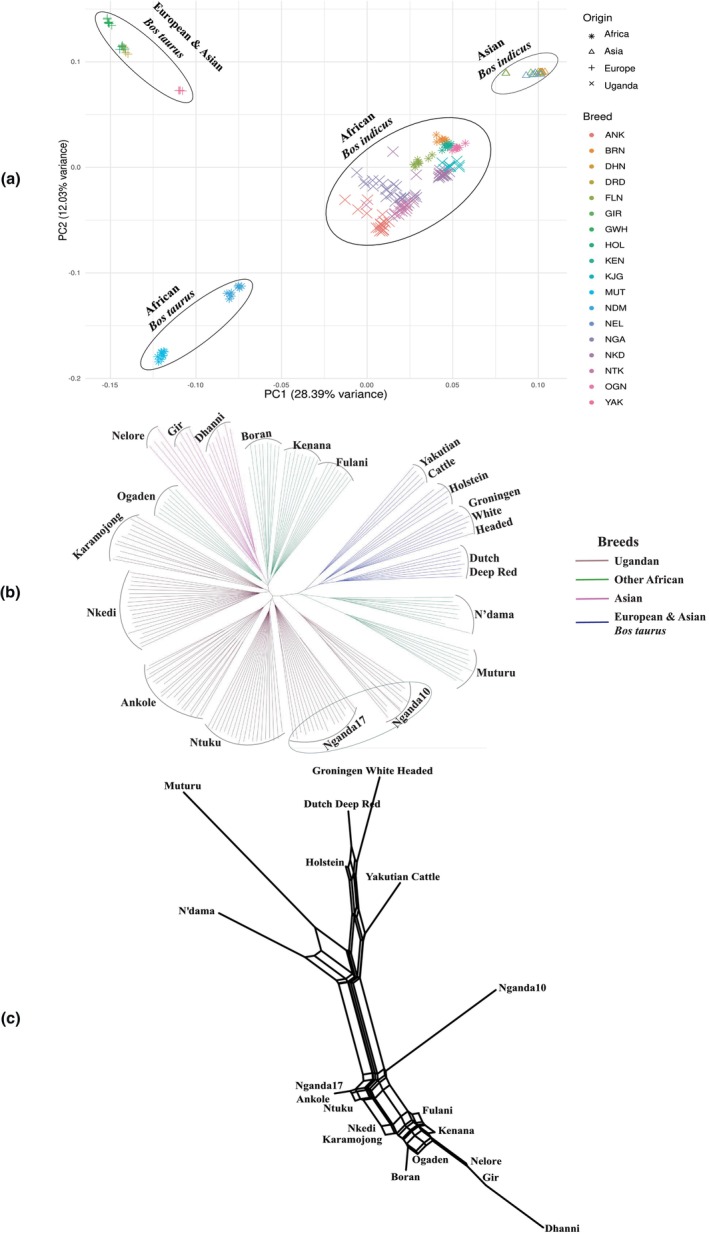
(a) Principal component (PC) analysis for all the 18 breeds. In the plot, clear clustering is seen for Asian, European, and African indicine and taurine breeds. Ugandan cattle can be seen as part of the African indicine cluster. (b) Neighbor joining phylogenetic tree for the 18 breeds. The Nganda breed is seen to form two distinct clusters in the phylogenetic tree. (c) NeighborNet plot derived from the *F*_ST_ matrix of the 18 breeds depicts major clusters of *Bos taurus* and *Bos indicus* breeds. Edges for the Nganda10 and Nganda17 are placed separately on the plot. Breed abbreviations are listed in Table 1.

Principal component analysis separated the 18 breeds into four clusters: Asian *B. indicus*, European and Asian *B. taurus*, African *B. taurus* (Muturu and N'dama) and a cluster of nine African *B. indicus* breeds (Figure 2a). Within the African indicine group, the Ugandan Sanga and Zenga (Ankole, Nganda and Ntuku) formed a visible subcluster, while the two Ugandan EASZ (Nkedi and Karamojong) subclustered with the other African indicine (Boran, Fulani, Kenana, and Ogaden).

### Phylogenetic clustering

The phylogenetic clustering by NJ clearly differentiated taurine and indicine breeds, with European and Asian taurine (Deep red, Groningen White Headed, Holstein, and Yakutian cattle) as well as the African taurine (N'dama and Muturu) clustered separately from the rest (Figure 2b). The Asian indicine breeds clustered with African indicine (Ogaden, Boran, Kenana, and Fulani), but were separate from the Ugandan breeds. Two of the Uganda breeds (Nkedi and Karamojong) belong to the EASZ category and clustered separately from the Sanga and Zenga breeds (Ankole, Ntuku, and Nganda). Interestingly, the phylogenetic tree (Figure 2b), showed a subgroup of the Nganda breed composed of 10 animals that clustered separately from other Nganda and Ugandan animals. These 10 animals clustered close to the taurine breeds.

A NeighborNet plot (Figure 2c) was created from the *F*_ST_ matrix (Table S5) of the 18 breeds. The plot separated all breeds into two major clusters composed of indicine and taurine breeds as had been observed in the NJ tree (Figure 2b). The cluster with taurine breeds had two subclusters of European, Asian and African taurine breeds, respectively. The indicine cluster had three subclusters: one for the Asian breeds and two for the African breeds. African Zebu breeds (including Nkedi and Karamojong breeds from Uganda) formed a separate subcluster from Ugandan Sanga and Zenga breeds (Ankole, Ntuku, and Nganda breeds). Notably, edges for Nganda10 and Nganda17 were distinctively placed on the plot.

### Admixture results

**FIGURE 3:**
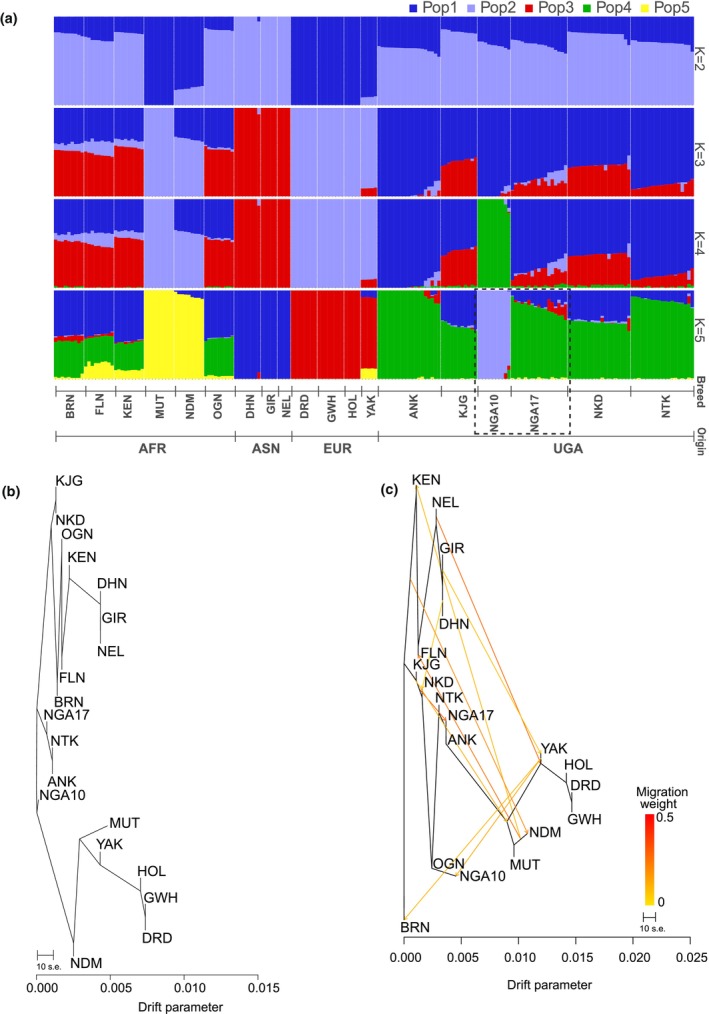
(a) Admixture plot for *K* values 2–5. The ideal predictive veracity is at *K* = 5. The distinct colors represent the proportion of each animal assigned to a given ancestry. The Nganda breed presents two different color patterns. (b) All breeds are grouped into three clusters by Treemix at zero migration events. (c) Several potential gene transfer events are seen in the plot at 10 migrations. Breed abbreviations are listed in Table 1.

The ideal model for inferring genetic composition across the 18 breeds was obtained at *K* = 5, which showed the lowest cross‐validation error (Figure S2a). The five ancestral populations were defined based on the highest ancestry proportions: Asian *B. indicus* (Gir, 100%), Uganda‐1 (Nganda, 36.89%), European taurine (Dutch Deep Red, 100%), Uganda‐2 (Ankole, 96.82%), and African taurine (Muturu, 100%; Figure 3a, Table S6A). Within Nganda cattle, the Uganda‐1 ancestry originated predominantly from the Nganda10 population (97.77%; Table S6B). The Asian *B. indicus* ancestry dominated Dhanni, Gir, and Nelore and was detectable in most breeds except European taurine and Muturu. The European ancestry was strongest in European breeds and present in several others, but absent in Gir, Nelore, Muturu, N'dama, and Ogaden. The Uganda‐2 ancestry characterized African *indicus* breeds but was absent in N'dama, Muturu, and all Asian and European breeds. The Uganda‐1 ancestry was shared with other African indicine but not with African taurine, Asian, or European breeds. The Muturu ancestry appeared in most African breeds except Nganda10 and was absent in Asian and European cattle.

At *K* = 5, Admixture suggested shared ancestry between African taurine breeds (Muturu and N'dama) and Yakutian cattle. However, patterns at lower *K* values (*K* = 2–4), the PCA (Figure 2a), and the NeighborNet plot (Figure 2c) indicate that Admixture probably assigned this shared ancestry, because the cattle populations in question lacked pure ancestry from any of the inferred clusters. Although the breeds in question are both taurine, they diverged centuries ago following the domestication events of cattle from the wild aurochs (Loftus et al., 1999) and currently inhabit geographically distant regions (West Africa and the Sakha Republic of Russia, respectively). Geneflow between these cattle populations is therefore highly unlikely.

**FIGURE 4:**
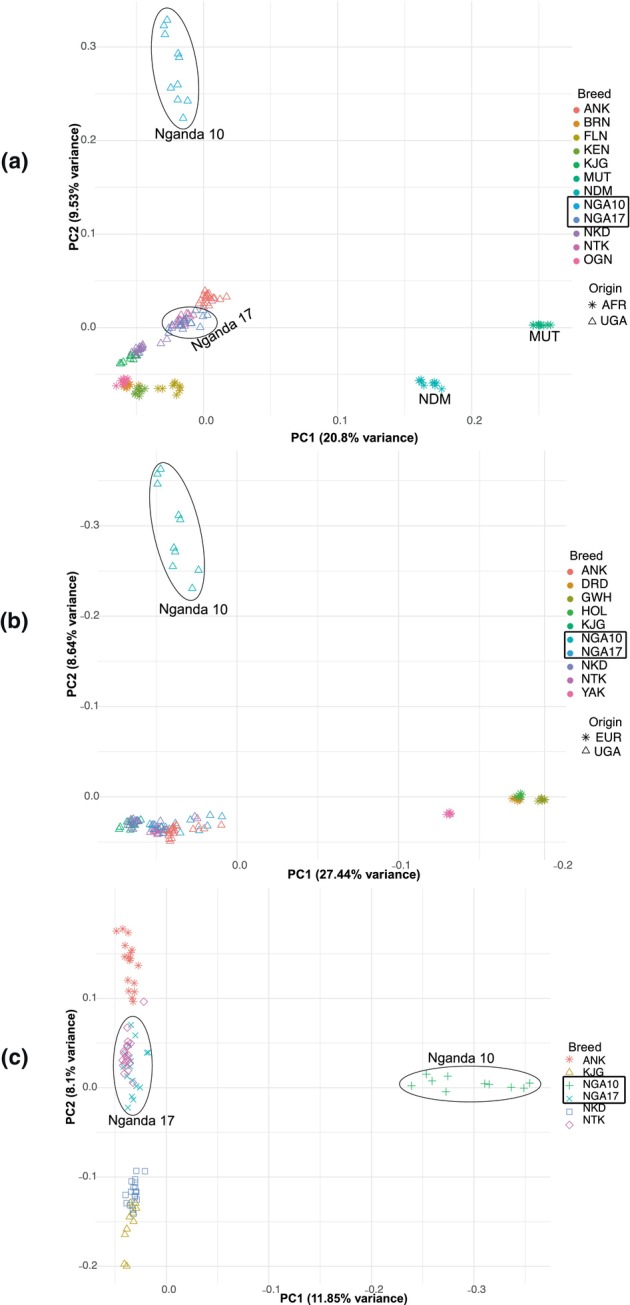
(a) Principal component (PC) analysis of Ugandan (UGA) and other African (AFR) breeds. The N'dama, Muturu, and a section of the Nganda cluster away from others. (b) PC analysis plot of European and Asian taurine (EUR) and Ugandan cattle shows a cluster of Nganda animals separating from other Ugandan cattle. (c) The PC analysis plot for 5 Ugandan breeds shows a subgroup of the Nganda breed composed of 10 animals (Nganda10) forming a separate cluster, away from the rest. As seen in all plots, the Nganda17 cattle are part of a cluster that includes the Ankole and Ntuku populations. Breed abbreviations are listed in Table 1.

As observed in the PCA for African and Ugandan breeds (Figure 4) and the NJ tree (Figure 2b), the same 10 Nganda animals that were distinct from the rest of the breed in earlier analyses showed the highest proportion (97.77% ± 0.05%) of the predicted Uganda‐1 (Pop2) ancestral population (Figure 3a, Table S6B).

### Sub‐clustering in the Nganda breed

To explore subgrouping within the Nganda breed, we used the population structure analysis to separate Nganda animals into two clusters, designated Nganda10 and Nganda17 according to sample size. In the subsequent sections of this study, these are considered as putatively distinct genetic populations. We performed three additional PCAs: two for Ugandan breeds with European and Asian taurine, and African breeds and the third exclusively featuring Ugandan breeds. All the three plots (Figure 4a–c) showed a section of the Nganda cattle (Nganda10) clustering away from the rest. The subclustering of the Nganda breed was consistent with grouping patterns observed in the NJ tree (Figure 2b), NeighborNet plot (Figure 2c), Admixture analysis (Figure 3a), and Treemix analysis (Figure 3b,c).

### Evolutionary relationships with Treemix

The ML tree without migration events, as modeled by Treemix, split the cattle populations into three clusters (Figure 3b): a taurine cluster (N'dama, Muturu, Yakutian cattle, Deep Red, Groningen White Headed, Holstein), an indicine/Zebu cluster (Karamojong, Nkedi, Ogaden, Kenana, Boran, Fulani, Dhanni, Gir, Nelore), and an intermediate Sanga/Zenga cluster (Nganda17, Ntuku, Ankole), with Nganda10 positioned between the Sanga/Zenga and taurine clusters. Incorporating 10 migration events (Figure 3c) resulted in a model that explained 99.8% of the observed genetic variance, highlighting the substantial contribution of gene flow to genetic relationships among the breeds.

### Genetic diversity

#### The genetic diversity parameters

**FIGURE 5:**
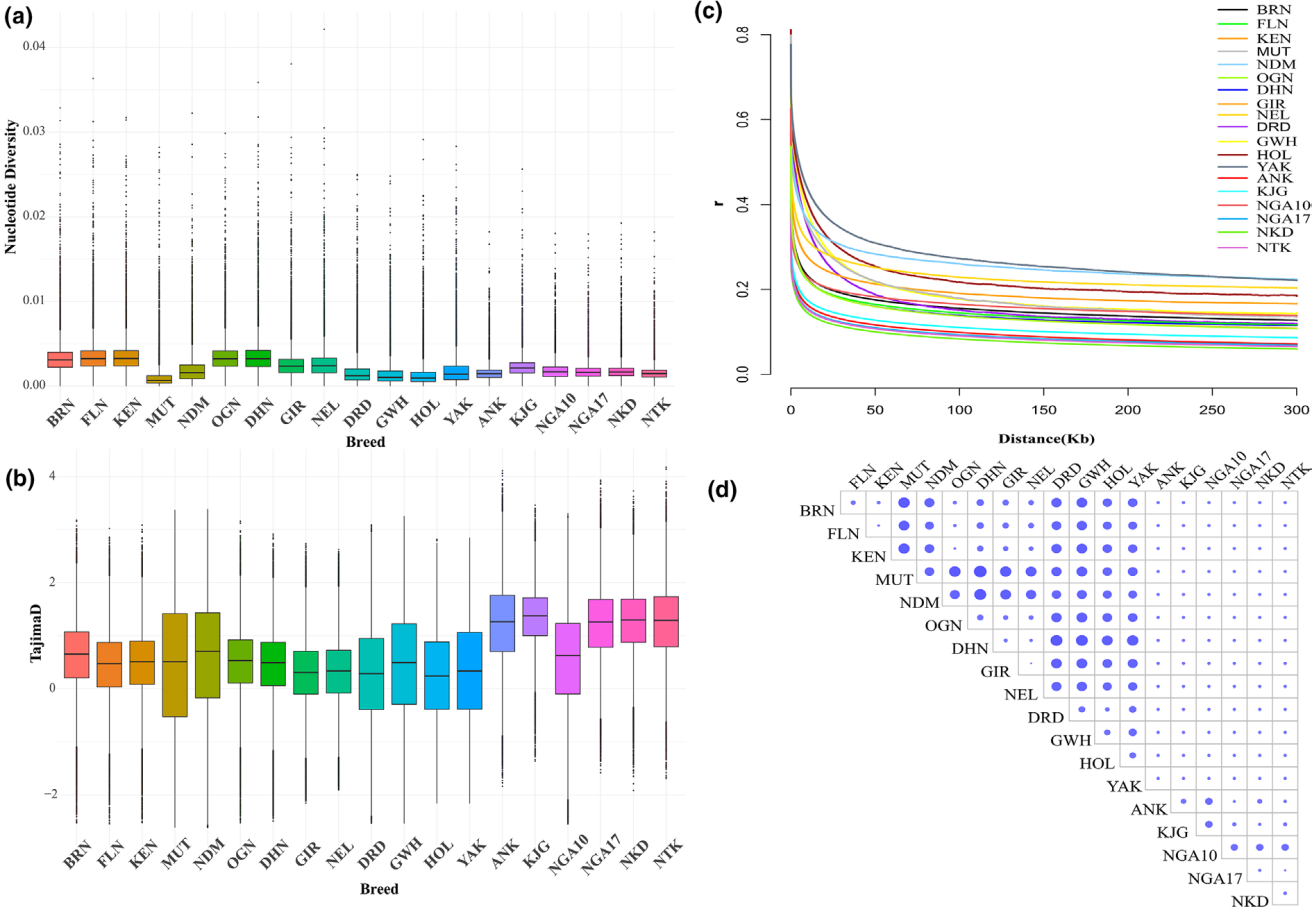
Plots of (a) nucleotide diversity values, (b) average Tajima's *D* statistic values, (c) decay of linkage disequilibrium, and (d) pairwise *F*_ST_ values as equivalent dot sizes across all the breeds. Breed abbreviations are listed in Table 1.

We assessed the different genetic diversity parameters based on the dataset of 22 455 225 high quality biallelic autosomal SNPs that had been used for population structure analysis. Nucleotide diversity analysis returned results (weighted by number of SNPs per window) ranging from 0.0027 ± 0.0011 (Ankole) to 0.0032 ± 0.0014 (Karamojong) among the Ugandan breeds and 0.0017 ± 0.0021 (Muturu) to 0.0026 ± 0.0019 (Yakutian cattle) among the rest of the breeds (Table S7). European breeds generally had lower nucleotide diversity values followed by Ugandan breeds while the Asian breeds had the highest (Figure 5a). The results are indicative of minimal sequence disparities and reduced genetic diversity among animals of a given breed. The lowest Tajima's *D* statistic values were observed in Yakutian cattle (0.2770 ± 1.0216) and the highest in Nkedi cattle (1.3344 ± 0.6049; Table S8). With the exception of the Nganda10 (0.5662 ± 0.9677), Ugandan breeds had higher average Tajima's *D* values compared to the rest (Figure 5b), indicating higher genetic diversity without extreme polymorphism within these breeds.

The observed heterozygosity (*H*_o_) was generally lower among the Ugandan breeds, ranging from 0.2291 ± 0.1344 (Ntuku) to 0.2745 ± 0.1783 (Nganda10) compared to the other breeds 0.2923 ± 0.1852 (Muturu) to 0.3587 ± 0.2060 (Yakutian cattle). Likewise, the expected heterozygosity (*H*_e_) was lower among the Ugandan breeds, ranging from 0.3078 ± 0.1475 (Nganda10) to 0.3398 ± 0.1257 (Karamojong). Overall, the *H*_o_ was lower than the *H*_e_ among the Ugandan breeds pointing to potential inbreeding (Chesnokov & Artemyeva, 2015). The homozygosity based inbreeding coefficient (*F*_HOM_) was higher among the Ugandan breeds ranging from 0.1089 ± 0.1206 (Nganda10) to 0.2658 ± 0.0212 (Ntuku). The rest of the breeds had *F*_HOM_ ranging from −0.0710 ± 0.0432 (N'dama) to 0.0681 ± 0.2330 (Gir). These high values among the Ugandan breeds suggest reduced genetic diversity, reduced heterozygosity, due to potential inbreeding (Kanaka et al., 2023), selection pressure or other genetic and environmental factors. The lowest average minor allele frequency among all the 18 breeds was observed in the Fulani breed (0.2192 ± 0.1387) while the highest was in Nelore (0.2666 ± 0.1283; Table S9).

The highest overall number of ROH (2748 segments, mean 144.63 ± 29.50), was observed in the Ntuku breed, but the highest mean number of ROH was in the Groningen White Headed (302.25 ± 78.93, 2418 segments; Table S10), with the majority being 0.5–1 Mb in size (Ntuku, 2077 segments and Groningen White Headed, 1500 segments; Figure S2b, Table S11a). The Yakutian cattle had the longest average ROH (1269.95 ± 70.62 kb) while Fulani had the shortest (634.69 ± 41.62 kb). Among the Ugandan breeds, Ntuku had the longest average ROH (893.32 ± 151.41 kb) whereas Karamojong had the shortest (671.83 ± 27.17 kb), with no segments above 5 Mb in length (Figure S2c, Table S11B). The average ROH‐derived inbreeding coefficient (*F*_ROH_) was lowest in Fulani (0.0101 ± 0.0098) and highest in Yakutian cattle (0.1381 ± 0.0121). Among the Ugandan breeds, the lowest average *F*_ROH_ was observed in the Karamojong (0.0173 ± 0.0051), while the highest was in the Ntuku cattle (0.0535 ± 0.0230; Table S10).

The fastest average decay of LD value was observed among the Ugandan cattle breeds ranging from 0.0821 (Nkedi) to 0.1094 (Karamojong) for different distances between markers (<10, <50, >50 kb, and overall) except for Nganda10 (0.1622). The highest overall values were in Yakutian cattle (0.2712), while Holstein had the highest at distances <10 (0.8106) and <50 (0.2264; Figure 5c, Table S12).

The average pairwise *F*_ST_ results were lower among the Ugandan breeds ranging from 0.006 (Karamojong vs. Nkedi) to 0.112 (Karamojong vs. Nganda10), with Nganda10 vs. Nganda17 having a value of 0.096. This finding highlighted restricted geneflow among the Ugandan cattle breeds. Generally, African breeds had higher *F*_ST_ values compared to the rest, with Boran vs. Muturu being the highest (0.339). A similar pattern was observed for values between taurine and indicine breeds (Figure 5d, Table S5).

## DISCUSSION

The aim of this study was to explore the population structure and genetic diversity of five native Ugandan cattle breeds. We analyzed whole genome sequences of 95 animals and combined our data with publicly available whole genome sequences of 97 animals in selected breeds originating from the rest of Africa, Asia, and Europe.

PCA results showed clear distinction between Asian *B. indicus*, European and Asian taurine, African taurine and African indicine breeds, which is consistent with previous reports (Friedrich et al., 2024; Gebrehiwot et al., 2020; Terefe et al., 2022; Tijjani et al., 2022). The NJ phylogenetic tree and NeighborNet plot supported the PCA findings and further revealed two subclusters for Ugandan cattle. These included one featuring EASZ (Karamojong and Nkedi) alongside other African Zebu breeds and the other having Sanga/Zenga (Ankole, Nganda, and Ntuku) breeds. The distinct clustering of Sanga/Zenga from other African Zebu has been previously observed with separation of the Ankole from other African zebu breeds (Kim et al., 2017; Nanaei et al., 2020; Taye et al., 2017; Tijjani et al., 2022, 2024). The ML tree modeled at 10 migration events showed geneflow from African taurine to African indicine cattle exemplified by the flow from N'dama to Fulani and Kenana. A similar pattern was previously reported for Sudanese cattle populations (Tijjani et al., 2022) and possibly shows flow of trypanotolerance variants from the trypanotolerant N'dama (Trail et al., 1989, 1990) into these African *B. indicus* breeds. Moreover, we observed strong geneflow from EASZ (Nkedi and Karamojong) to Nganda (Nganda17), which is a known event given that Nganda cattle are crosses of EASZ and Sanga cattle (Rege & Tawah, 1999).

### European ancestry introgression into native Ugandan cattle

Our admixture results showed the introgression of European ancestry into several Ugandan cattle breeds, peaking in the Nganda17. Similar introgression of European ancestry into African cattle has been reported for the Ankole (Nanaei et al., 2020) and Kenyan EASZ (Mbole‐Kariuki et al., 2014). The European ancestry introgression into Ugandan breeds could be due to the use of exotic bulls or their semen, given the low numbers of bulls (18.2% of adult cattle) in the Ugandan herd (UBOS, 2024). Notably, the farm manager at the Nganda10 sampling site noted the recent use of a non‐native bull in the herd (personal communication) due to the absence of a native male of breeding age on the farm. This non‐native Nganda bull could have sired the animals that were detected in our admixture analysis with an observed European ancestry within the Nganda10 population. Similar observations were made for the other Ugandan breeds except Karamojong cattle, highlighting recent cross breeding events in the Ugandan native herd. The absence of this observation in the Karamojong animals is probably due to the pastoral husbandry practices of the Karamojong people, which are unfavorable for exotic and crossbred animals. Coincidentally, the number of exotic/crossbred cattle in the region was reported to be below 0.5% of the country's total exotic/crossbred herd in 2021 (UBOS, 2024). Several African countries have policies inclined towards exotic and crossbred animals (Nyamushamba et al., 2016). It is imperative that authorities in such countries avert the loss of valuable animal genetic resources possessed by parent native breeds through intensification of efforts for the collection and storage of genetic materials such as semen and embryos in gene banks. Moreover, up to 61.54% of the local breeds and 86.21% of transboundary domestic animal breeds in Uganda have no genetic materials stored in gene banks for reconstitution (FAO_DAD‐IS, 2024). Such conservation efforts on the continent could be spearheaded by the five Regional Animal Gene Banks established by the African Union in West Africa (Burkina Faso), Southern Africa (Botswana), Eastern Africa (Uganda), Central Africa (Cameroon), and North Africa (Tunisia).

### Breed differentiation and genetic structure

The highest average Tajima's *D* statistic values (Table S8) were observed among Ugandan breeds. Nkedi cattle exhibited the highest (1.33 ± 0.61) while Nganda10 showed the lowest (0.62 ± 0.92), suggestive of population bottlenecks or balancing selection to maintain important genetic variants. Such variants may include those involved in host–pathogen interactions, particularly in response to disease challenges such as tick‐borne infections and trypanosomiasis (Byaruhanga et al., 2017; Kasaija et al., 2021; Kizza et al., 2021; Mandela et al., 2020). The highest average number of ROH was observed among Ugandan cattle breeds, characterized by increased frequency of short (0.5–1 Mb) and medium (1–5 Mb) segments, whereas other African and European (and Asian taurine) breeds exhibited higher frequencies of segments in the long (>5 Mb) category. Long ROH segments generally indicate recent inbreeding, while short segments point to ancient inbreeding (Broman & Weber, 1999). Ugandan breeds showed moderate *F*_ROH_ values, while the highest were observed in European breeds (and Asian taurine) and the lowest in Asian *B. indicus* breeds. These patterns probably result from intense artificial selection in European breeds and uncontrolled inbreeding in Ugandan breeds. The reduced genetic diversity and inbreeding observed in Ugandan cattle may be attributed to the traditional grazing practices of native cattle keepers. These practices involve herding mixed‐age and mixed‐sex animals in communal settings (Wolff et al., 2019), which complicates controlled mating. The situation is further exacerbated in agro‐pastoral communities where most breeds except Nganda are reared. In these areas, cattle may travel up to 200 km in search of water and pasture, spending nearly 2 months under the care of herdsmen (Ssekibaala et al., 2024), who are often unaware of the cattle owners' breeding objectives.

Breed differentiation as determined by *F*_ST_ showed the greatest divergence among African breeds (excluding Ugandan breeds), followed by European (and Asian taurine) and Asian *B. indicus* breeds, with Ugandan cattle exhibiting the lowest average values. The high average *F*_ST_ values observed in African breeds are probably due to the presence of both taurine (N'dama and Muturu) and indicine breeds within this group. Conversely, the low *F*_ST_ values among the Ugandan breeds could be explained by the complex genetic relationships that exist between them. The Ankole and Ntuku are both Sanga breeds, with Ntuku considered a strain of Ankole. On the other hand, the Nkedi and Karamojong are both EASZ while the Nganda are crosses of Zebu and Sanga (Zenga). Specifically, Nganda cattle have been described as crosses between the Ankole and Nkedi (FAO, 1957; Rege & Tawah, 1999).

### Nganda subpopulation divergence

Our admixture, phylogenetic, and NeighborNet analysis pointed to a potential split of one of the Ugandan breeds (the Nganda) into two strains. PCA of the Nganda cattle with the other African breeds and later with only the Ugandan breeds supported this potential split. A closer look at the sample metadata revealed that animals in this splinter group (Nganda10) originated from a single sampling site at a government conservation farm. According to the genetic diversity parameters, the genetic distance between the two breeds estimated by *F*_ST_ calculation was 0.096, a value higher than 0.009 for Sanga breeds (Ankole vs. Ntuku) and 0.006 for the EASZ breeds (Nkedi vs. Karamojong). Moreover, Nganda10 showed higher values of LD (0.16) than the Nganda17 (0.09) and lower nucleotide diversity (0.0027) than Nganda17 (0.0029). The Nganda10 subgroup had a slightly lower *F*_ROH_ (0.0414) and *F*_HOM_ (0.0189) compared to the Nganda17 (0.0446 and 0.2527, respectively). The observed splitting of the Nganda breed could be attributed to the presence of an initial Nganda population, and a population arising from breeding between the Nganda and other breeds. Our findings are not in isolation, as similar breeding trends leading to hybrid populations were previously reported for the Ankole and Zebu resulting in the emergence of Nganda and Nyoro hybrids in central Uganda (Epstein, 1957). Similar observations were reported for subpopulations of the same cattle breed; the Leiqiong cattle of China (Guo et al., 2025), and among Tibetan cattle (Lei et al., 2006).

The knowledge of previous historic changes undergone by populations is essential in species conservation efforts (Eizaguirre & Baltazar‐Soares, 2014). To confirm our hypothesis, we engaged a researcher who was involved in the establishment of this Nganda10 herd at the government farm. It was revealed that the herd was put together between 1997 and 1998 as a nucleus herd for the Nganda breed, following conservation efforts by the Uganda National Agricultural Research Organization to conserve native cattle breeds. At the time, such nucleus herds were also established for six other breeds including the Teso, Nkedi, Kigezi, Ankole, Lugware, and Karamojong. These conservation animals were kept at the National Livestock Resources Research Institute farm in the eastern district of Tororo. It was mentioned that the conserved animals for the Nganda breed (Nganda10) were obtained from northern districts of the central region of Uganda. This area lies in the cattle corridor where animal husbandry practices are majorly pastoral and agro‐pastoral (Kasaija et al., 2021). On the contrary, animals from which the Nganda17 samples were collected are native to the southern part of the central region. This area lies in the agro‐ecological zone around Lake Victoria, where tethering of livestock is a common practice (Kabi et al., 2016). Similar to our narrative, geographical and climatic differences were previously reported to influence the genetic diversity of native cattle and sheep breeds in Italy (Senczuk et al., 2022) while in Mexico, Criollo Cattle from the Temoris region were reportedly distinct from Criollo cattle from five other regions of the country (Russell et al., 2000).

The above narrative presents a complex challenge to animal genetic resource conservation efforts in regions where focus animals span large geographical areas with various ecological conditions. With whole genome sequence data, we were able to discern differences between these two subpopulations of the same breed (Nganda). Observation of such changes in the genetic status of populations has been underscored as key to informing policy and management actions as well as perspectives of biologists involved in conservation (Eizaguirre & Baltazar‐Soares, 2014). One such action could be the maintenance of two separate conservation herds of Nganda breed, with cattle populations from the northern and southern parts of the central region of the country.

## CONCLUSION

In this study, we have used whole genome sequence data to provide novel and comprehensive insights into the genetic diversity, and population structure of selected native cattle breeds from Uganda. Findings from our study are in line with previous reports about presence of three different indigenous cattle types (the Sanga, the Zebu, and Zenga) in Uganda. Notably, we report the existence of two genetically distinct populations of Nganda cattle. Moreover, our results provide unique perspectives into the breeding situation of native cattle populations in the country highlighting the admixture of exotic genetic resources into indigenous cattle. Several interventions to contain this admixture issue will be required to foster the maintenance of the genetic integrity of native animals amid threats from crossbred and exotic animals and their genetic material. Our study lays a foundation for future research on the genetic characteristics of native cattle for further documentation of the country's animal genetic resources. Our observations need to be investigated further within the Nganda and other native breeds to better understand their genomic architecture and identify the underlying signatures of selection and adaptation to their unique environments.

## AUTHOR CONTRIBUTIONS

**Rodney Okwasiimire:** Investigation; writing – original draft; writing – review and editing; visualization; validation; methodology; formal analysis; data curation; conceptualization; resources. **Donald R. Kugonza:** Conceptualization; funding acquisition; validation; writing – review and editing; supervision; resources. **Daniil Ruvinskiy:** Writing – review and editing; validation; investigation; visualization. **Melak Weldenegodguad:** Writing – review and editing; validation; investigation; visualization. **Nasser Ghanem:** Resources. **Mahlako L. Makgahlela:** Conceptualization; funding acquisition; validation; writing – review and editing; resources. **Catarina Ginja:** Conceptualization; funding acquisition; validation; writing – review and editing; resources. **Richard P. M. A. Crooijmans:** Conceptualization; funding acquisition; validation; writing – review and editing; project administration; resources. **Juha Kantanen:** Conceptualization; funding acquisition; writing – review and editing; validation; supervision; methodology; resources. **Pekka Uimari:** Supervision; writing – review and editing; validation; methodology; conceptualization; funding acquisition. **Kisun Pokharel:** Methodology; validation; visualization; writing – review and editing; supervision; investigation.

## FUNDING INFORMATION

This work was funded under the OPTIBOV (Genetic characterization of cattle populations for optimized performance in African ecosystems) project, part of the Long‐term EU‐Africa Research and Innovation Partnership on Food and Nutrition Security and Sustainable Agriculture (LEAP‐Agri) of the European Union's Horizon 2020 research and innovation program (LEAP‐Agri‐326, grant number: 727715). Co‐funding was provided by the Government of the Republic of Uganda through the Science Technology and Innovation (STI) Secretariat (grant number: MoSTI/LEAP‐11). Rodney Okwasiimire acknowledges funding from the University of Helsinki as a salaried doctoral researcher.

## CONFLICT OF INTEREST STATEMENT

The authors declare no conflict of interest.

## ETHICS STATEMENT

The study samples were collected following approval from the Makerere University School of Biosecurity, Biotechnical and Laboratory Sciences (SBLS) Higher Degrees Research and Ethics Committee (Reference number: SBLS/HDRC/20/001).

## Supporting information


Figure S1.

Figure S2.

Table S1.

Table S2.

Table S3.

Table S4.

Table S5.

Table S6.

Table S7.

Table S8.

Table S9.

Table S10.

Table S11.

Table S12.

## Data Availability

The raw sequence data analyzed in this study are available in the European Nucleotide Archive under the project accession number PRJEB90914. The specific sample accession numbers are ERS25076563–ERS25076573 (Karamojong), ERS25076574–ERS25076592 (Ntuku), ERS25076593–ERS25076599 (Nganda17), ERS25076600–ERS25076609 (Nganda10), ERS25076610–ERS25076619 (Nganda17), ERS25076620–ERS25076638 (Ankole), and ERS25076639–ERS25076657 (Nkedi).
