# Supplementary material for: Genomic insights into the population structure and genetic diversity of Ugandan indigenous cattle
Source: Anim Genet. 2025 Oct 27;56(6):e70050. doi: 10.1111/age.70050 (PMC12559783; doi:10.1111/age.70050)
Supplement: Supplementary file 1 — Figure S1. [file AGE-56-0-s012.pdf]

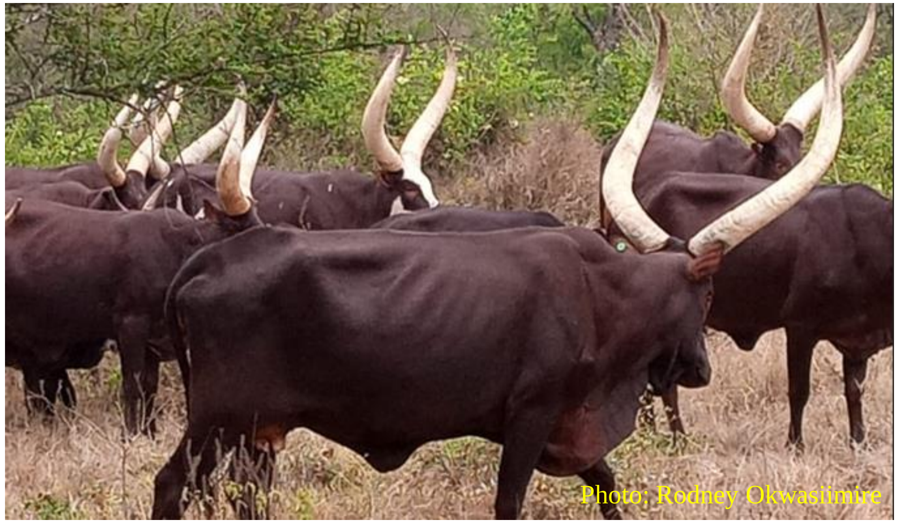
Photo: Rodney Okwasiimire

(a)

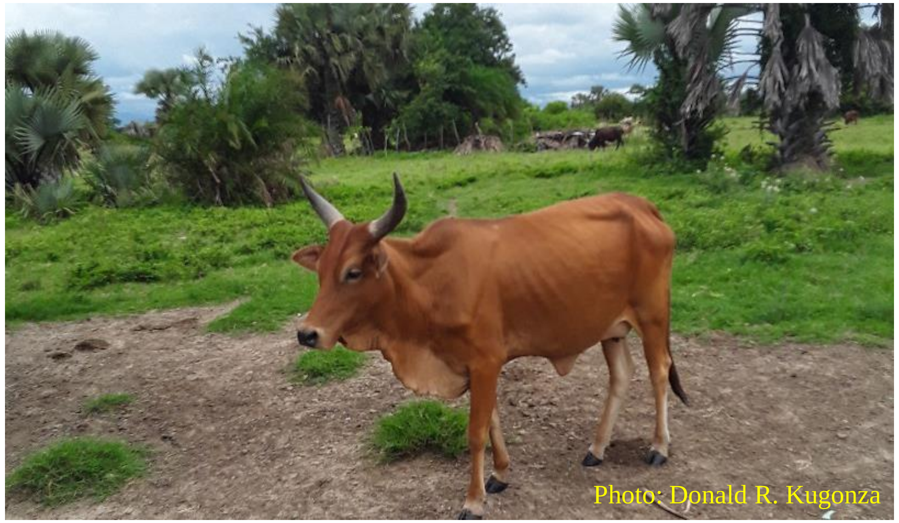
Photo: Donald R. Kugonza

(b)

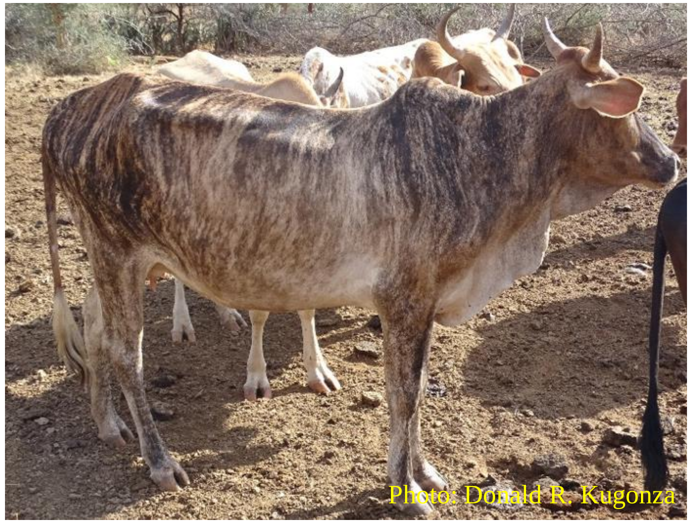
Photo: Donald R. Kugonza

(c)

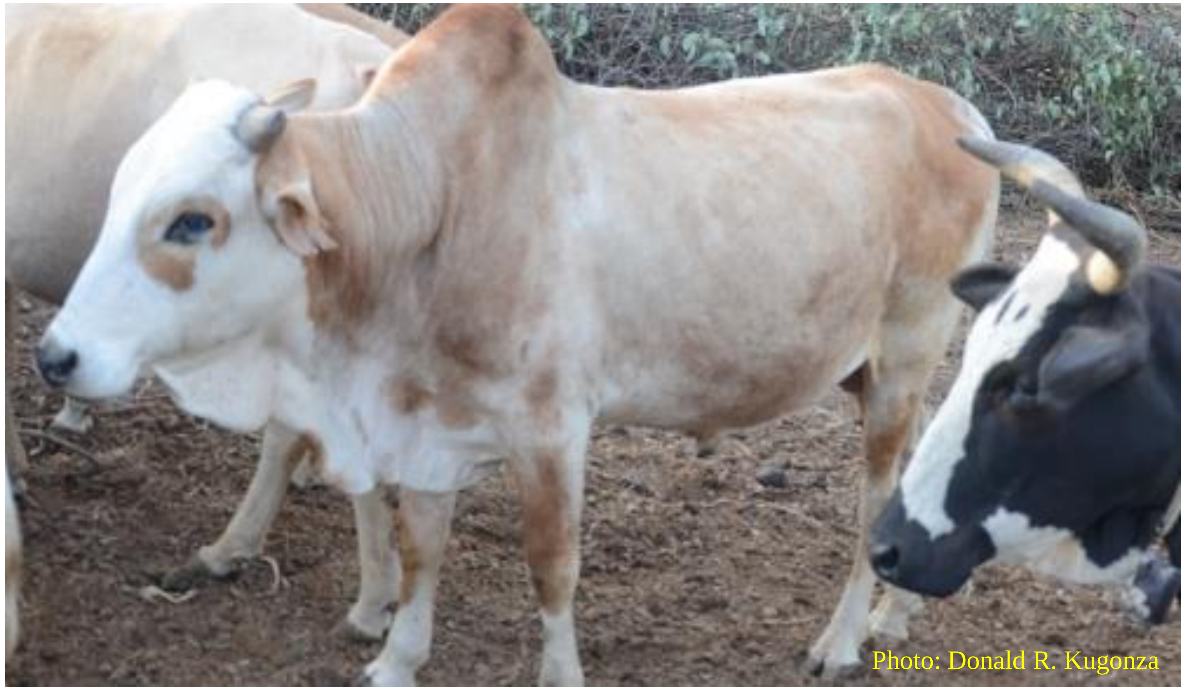
Photo: Donald R. Kugonza

(d)

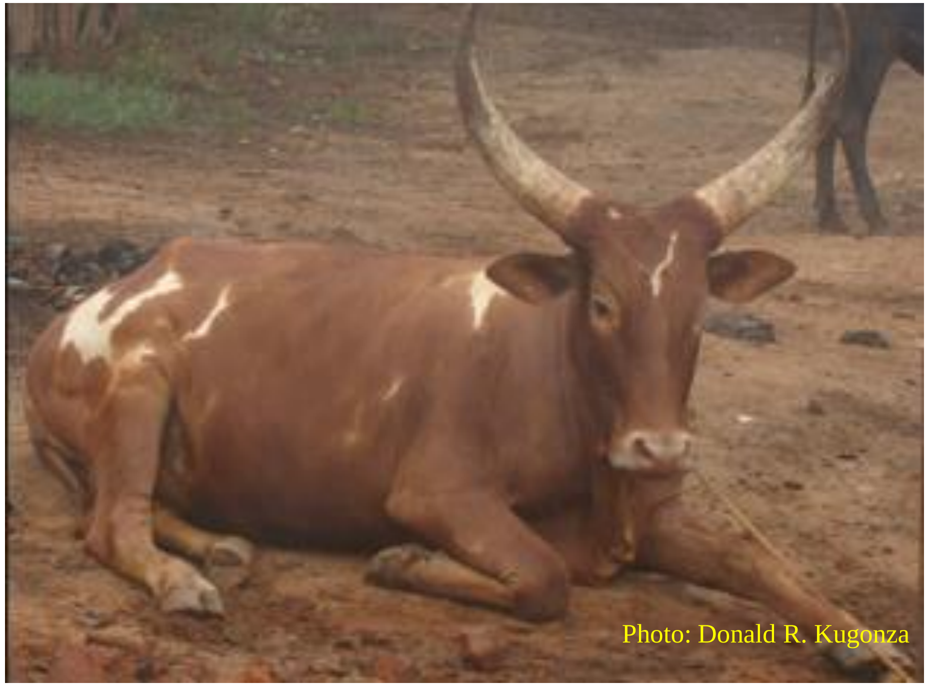
Photo: Donald R. Kugonza

(e)
