# Supplementary figures and images for: Genomic insights into the population structure and genetic diversity of Ugandan indigenous cattle
Source: Anim Genet. 2025 Oct 27;56(6):e70050. doi: 10.1111/age.70050 (PMC12559783; doi:10.1111/age.70050)

(a)

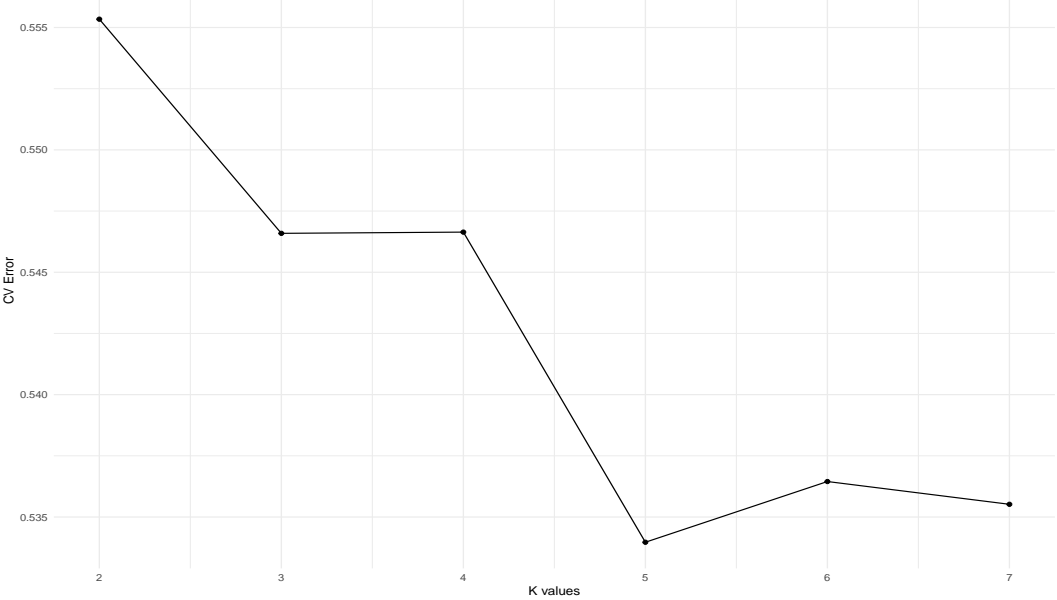

(b)

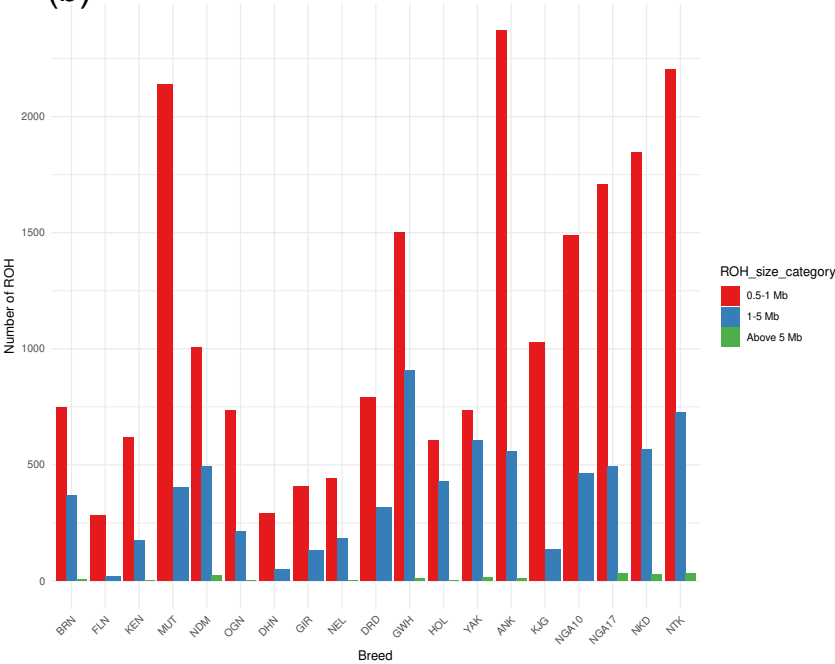

(c)

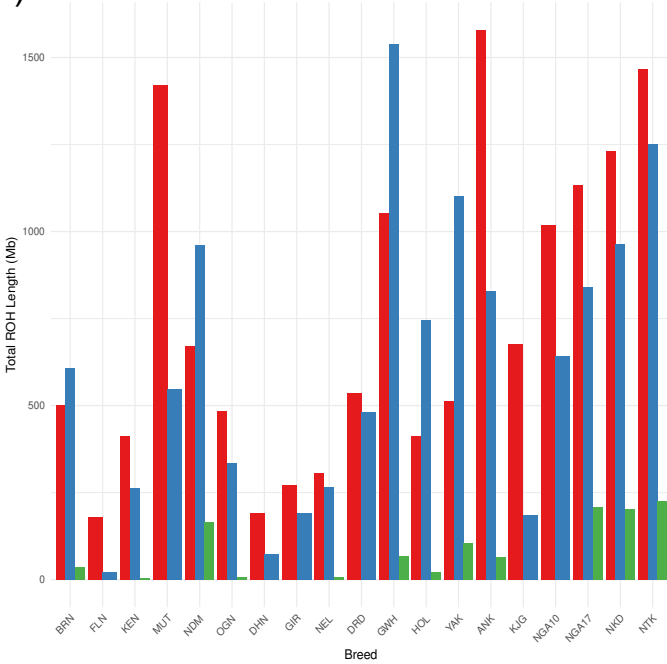

Supplement: Supplementary file 2 — Figure S2. [file AGE-56-0-s014.pdf]
